# Ribo-Seq and RNA-Seq of *TMA46* (*DFRP1*) and *GIR2* (*DFRP2*) knockout yeast strains

**DOI:** 10.12688/f1000research.74727.1

**Published:** 2021-11-16

**Authors:** Artyom A. Egorov, Desislava S. Makeeva, Nadezhda E. Makarova, Dmitri A. Bykov, Yanislav S. Hrytseniuk, Olga V. Mitkevich, Valery N. Urakov, Alexander I. Alexandrov, Ivan V. Kulakovskiy, Sergey E. Dmitriev

**Affiliations:** 1 Phystech School of Biological and Medical Physics, Moscow Institute of Physics and Technology (State University), Dolgoprudny, 141700, Russian Federation; 2 Belozersky Institute of Physico-Chemical Biology, Lomonosov Moscow State University, Moscow, 119234, Russian Federation; 3 Sirius University of Science and Technology, Sochi, 354340, Russian Federation; 4 Faculty of Bioengineering and Bioinformatics, Lomonosov Moscow State University, Moscow, 119234, Russian Federation; 5 Engelhardt Institute of Molecular Biology, Russian Academy of Sciences, Moscow, 119991, Russian Federation; 6 Department of Chemistry, Lomonosov Moscow State University, Moscow, 119234, Russian Federation; 7 Bach Institute of Biochemistry, FRC of Biotechnology of the Russian Academy of Sciences, Moscow, 119071, Russian Federation; 8 Institute of Protein Research, Russian Academy of Sciences, Pushchino, 142290, Russian Federation

**Keywords:** Transcriptome, translatome, ribosome profiling, ribosome stalling, ribosome collision, Saccharomyces cerevisiae, TMA46, GIR2, GCN1/GCN20, eIF2A, YGR054W, STM1, PUB1

## Abstract

In eukaryotes, stalled and collided ribosomes are recognized by several conserved multicomponent systems, which either block protein synthesis *in situ *and resolve the collision locally, or trigger a general stress response. Yeast ribosome-binding GTPases RBG1 (DRG1 in mammals) and RBG2 (DRG2) form two distinct heterodimers with TMA46 (DFRP1) and GIR2 (DFRP2), respectively, both involved in mRNA translation. Accumulated evidence suggests that the dimers play partially redundant roles in elongation processivity and resolution of ribosome stalling and collision events, as well as in the regulation of GCN1-mediated signaling involved in ribosome-associated quality control (RQC). They also genetically interact with SLH1 (ASCC3) helicase, a key component of RQC trigger (RQT) complex disassembling collided ribosomes. Here, we present RNA-Seq and ribosome profiling (Ribo-Seq) data from *S. cerevisiae* strains with individual deletions of the *TMA46 *and *GIR2 *genes. Raw RNA-Seq and Ribo-Seq data as well as gene-level read counts are available in NCBI Gene Expression Omnibus (GEO) repository under GEO accession GSE185458 and GSE185286.

## Introduction

Here, we present Ribo-Seq and RNA-Seq data for *S. cerevisiae* strains lacking translation-associated proteins Tma46 and Gir2, as well as for the wild type BY4742 parent strain. Tma46 and Gir2 are yeast orthologs of two mammalian DRG family regulatory proteins: DFRP1 and DFRP2, respectively. ^1^^–^^3^ Mammalian DFRP1 and DFRP2 are binding partners of two closely related proteins, developmentally regulated GTPases DRG1 and DRG2, ^1^^,^^4^ while yeast Tma46 and Gir2 dimerize with their orthologs, the ribosome-binding GTPases Rbg1 and Rbg2, respectively. ^2^^,^^3^^,^^5^ Thus, in both yeast and mammals, two distinct heterodimers exist, RBG1•TMA46 (DRG1•DFRP1) and RBG2•GIR2 (DRG1•DFRP1), although under some conditions RBG1 may interact with GIR2 as well. ^5^ The RBG1 (DRG1) containing complexes associate with mono- and polysomes. ^2^^,^^3^^,^^5^^–^^7^ Using 5P-Seq, it was recently shown that RBG1•TMA46 promotes efficient translation in yeast, alleviating ribosome pausing at Arg/Lys-rich regions. ^7^ In contrast, the RBG2 (DRG2) containing dimers are not bound to ribosomes under normal conditions. ^3^^,^^6^ However, they are also clearly related to translation, as GIR2 interacts with the ribosome-bound GCN1, and RBG2•GIR2 is responsible for efficient cell growth under amino acid starvation. ^5^^,^^8^ GCN1 is a large protein necessary for activation of GCN2, the evolutionary conserved eIF2 kinase. ^9^ Recently, the RBG2•GIR2 complex was detected on the leading stalled ribosome on the Cryo-EM reconstruction of a GCN1-disome complex. ^10^ These results suggest that GIR2 is a physical linker between RBG2 and GCN1 and that this interaction could prevent excessive activation of the GCN2 pathway upon incidental ribosome stalling.

Interestingly, neither the yeast *rbg1Δ* or *rbg2Δ* knockout strains nor the double *rbg1Δrbg2Δ* mutants display any defects in translation or cell growth. ^3^ However, a genetic screen for triple synthetic interaction demonstrates that RBGs have redundant function with SLH1, ^3^ an RNA helicase involved in ribosome-associated quality control (RQC). SLH1 is an ortholog of mammalian ATCC3, a component of the ASC-1 complex that disassembles collided ribosomes (see 11 and references therein).

Taken together, the above data suggest that the RBG1•TMA46 (DRG1•DFRP1) and RBG2•GIR2 (DRG1•DFRP1) complexes play a role in elongation processivity and resolution of ribosome stalling and collision events, as well as in control of GCN1-mediated signaling accompanying these processes. However, many questions remain unanswered. In particular, the individual roles of the two distinct complexes are still unclear. To improve our understanding of their functions, we systematically characterized translational defects in *S. cerevisiae* strains with individual deletions of the *TMA46* or *GIR2* genes using ribosome profiling. ^12^ We present RNA-Seq and Ribo-Seq data for the yeast *tma46Δ* and *gir2Δ* knockout strains. For comparison, we also provide corresponding data for three strains bearing deletions of other translation-related genes: *STM1*, *PUB1* and *YGR054W* (encoding translation factor eIF2A), as well as for the wild type BY4742 parent strain. Raw sequencing data are available online in the NCBI Gene Expression Omnibus (GEO accession: GSE185458 and GSE185286).

## Materials and methods

### Yeast strains, cell maintenance, RNA-Seq and Ribo-Seq library preparation

RNA-Seq and Ribo-Seq сDNA libraries were prepared from total RNA samples or ribosome-bound RNA samples, respectively, for the wild-type BY4742 ( *MATα his3Δ1 leu2Δ0 lys2Δ0 ura3Δ0*) yeast strain and five knockout strains. The data were obtained in two independent series. The first one included the wild-type BY4742 yeast strain ( *wt*) and strains with individually deleted *YGR054W* (eIF2A), *STM1*, and *TMA46* genes, which were obtained from the Yeast Gene Deletion Collection. ^13^ The second one included the wild-type BY4742 yeast strain and strains lacking *GIR2* and *PUB1*, which were created during this study. KanMX disruption cassettes were obtained via polymerase chain reaction (PCR) using the primers ( *GIR2 -* 5′-CAATTGTAAAATCAGCAGGCA-3′; 5′-AAACTTGTCTATTTCCTTCTTC-3′; *PUB1 -* 5′-TTGTCCTTCATTTTCCTCTCGT-3′; 5′-AGGCCCTTTTATTTTTCGAGC-3′) and genomic DNA of the corresponding deletion collection strains as a template. Phusion HF polymerase (TFS F-530L) was used as recommended by the supplier. PCR protocol: 95°С – 5 min, [95°С – 30 sec, 64°С – 30 sec, 72°С – 60 sec] x 28 cycles, 72°С – 90 sec. The resulting cassettes were used to transform the BY4742 strain. The correctness of the integration was checked using pairs of verification primers ( *GIR2*-5′-GAAAAAGAAAGAAGAAAAATTTGGG-3′; *PUB1-*5′-ACGACCACAAAGGATCCAGGGCTT-3′; Universal primer inside the KanMX cassette - 5′-CTGCAGCGAGGAGCCGTAAT-3′).

Here we focus on *wt*, *tma46Δ*, and *gir2Δ* strains. The data from the other strains were used to correct for batch effects within each series. The libraries were sequenced, resulting in 31 RNA-Seq and 28 Ribo-Seq data sets, including 18 RNA-Seq and 16 Ribo-Seq data sets for *wt*, *gir2Δ*, and *tma46Δ* strains. Supplementary Table 1 in the *Extended data* ^14^ summarizes information about the sequencing experiments.

The experimental procedure followed the ribosome profiling protocol described in. ^15^ Briefly, yeast cells were grown to exponential phase (OD = 0.5-0.6) in yeast extract peptone dextrose (YPD) media (1% yeast extract, 2% peptone, 2% glucose). Cells were harvested by filtration, scraped into liquid nitrogen, and ground using a liquid nitrogen-cooled mortar and pestle with drop-by-drop addition of polysome lysis buffer (20 mM Tris-HCl pH 8.0, 140 mM KCl, 1.5 mM MgCl _2_, 0.1 mg/ml cycloheximide, 1% Triton). Cell lysates were clarified by two sequential centrifugation steps - 3000g, 5 minutes, 4°С, and 20000g, 10 minutes, 4°С. The cell lysate was partially used for mRNA isolation using oligo (dT) beads. Another portion was treated with ribonuclease I for polysome disassembly and applied to a linear 10-50% sucrose gradient in fractionation buffer (20 mM Tris pH 8.0, 140 mM KCl, 15 mM MgCl _2_, 1 mM DTT, 0.1 mg/ml cycloheximide, 1% Triton) and separated on a SW-41 rotor (Beckman) at 35000 rpm, 3 hours, 4°С. Subsequently, ribosome-bound RNA fragments were collected from the monosome fraction. Ribosome-bound RNA was isolated using acidic-phenol extraction. Further Ribo-Seq and RNA-Seq library preparations were performed as described previously. ^12^

### Sequencing data processing and analysis

Reads were trimmed using cutadapt v. 2.10 ^16^ with the following parameters for RNA-Seq (-a AGATCGGAAGAGCACACGTCTGAACTCCAGTCAC –minimum-length 20 -q 20) and Ribo-Seq samples (-a CTGTAGGCACCATCAATAGATCGGAAGAGCACACGTCTGAACTCCAGTCAC –trimmed-only -q 20). Additionally, for Ribo-Seq, the reads were deduplicated with seqkit rmdup v. 0.10.1, ^17^ and unique barcodes were then removed with cutadapt v. 2.10 (-q 20 –minimum-length 20 -u -4). Afterwards, reads were aligned against eukaryotic rRNA sequence set obtained from silva-euk ^18^ and rfam ^19^ databases using bowtie2 v. 1.2.3. ^20^ Only unmapped non-rRNA reads were used in the further analysis. Read mapping and counting against the Saccharomyces_cerevisiae.R64-1-1.95 (Ensembl) ^21^ genome assembly was performed with STAR v. 2.7.9a. ^22^ We estimated the position of the P-site for each dataset from the 5′ end of the reads on the basis of the length of each footprint using plastid v0.5.1. ^23^ Fraction of reads in each phase and read length distribution were also obtained with plastid, see Figure S1 in the *Extended data.* ^24^ The results show that almost 90% 28nt reads are in 0 phase through the annotated coding sequences (CDSs). Then we produced BedGraph profiles from SAM data with samtools v. 1.10 ^25^ and bedtools v2.27.1. ^26^ Coverage profiles were normalized using normalization factor and library size estimates from differential expression analysis (see below) separately for each bedGraph profile. Finally, we visualized coverage tracks in the modified genomic loci using svist4get. ^27^ Figure S2 in the *Extended data* ^24^ shows that the read counts originating from the mRNA encoded by the knockout gene in the corresponding strain are negligible. The coverage of the neighboring genes remains unaltered, i.e. there are no indications of the so-called neighboring gene effect (NGE). ^28^

### Differential expression and Gene Ontology (GO) enrichment analysis

**Figure 1.**
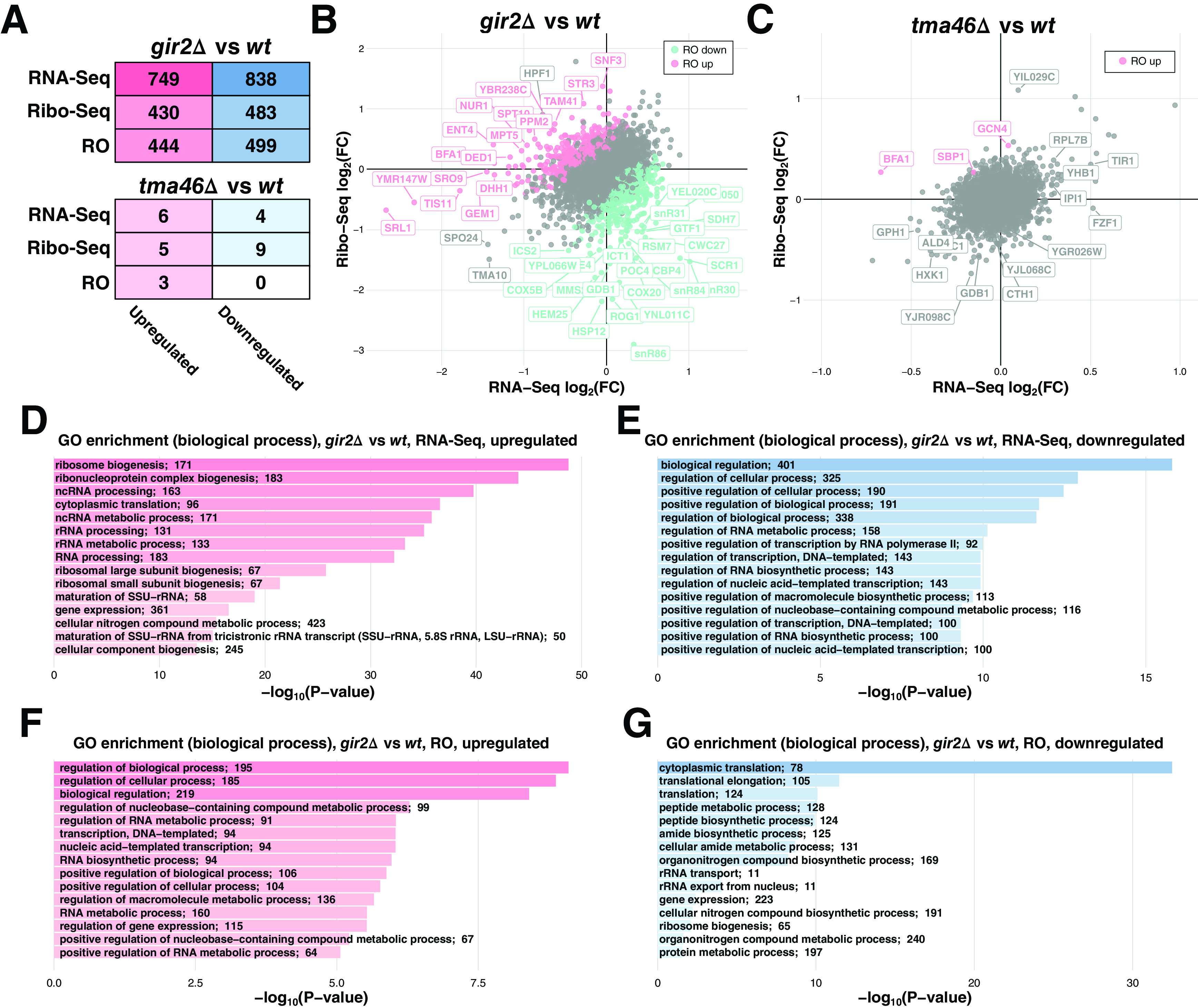
Differential gene expression upon deletions of *GIR2* and *TMA46.* (A) Total number of differentially expressed genes (passing FDR < 0.05) in each test. (B) and (C) Scatter plots illustrating expression changes at the level of transcription (X-axis, RNA-Seq) and translation (Y-Axis, Ribo-Seq) in the *gir2Δ* and *tma46Δ* strains, respectively. Translationally upregulated or downregulated (FDR < 0.05) genes marked in pink/blue, respectively. (D-G) Results of Gene Ontology (GO) enrichment analysis of RNA-Seq (D-E) and ribosome occupancy (RO) (F-G) considering upregulated (D, F) and downregulated (E, G) genes in *gir2Δ* vs. *wt* comparison. The numbers of differentially expressed genes with particular GO terms are shown in labels. X-axes show the enrichment P-value in log-scale.

Statistical analyses were performed in R v. 4.1.2 using edgeR Bioconductor package. ^29^ As mentioned above, the data were produced in two independent series which were analyzed separately. Genes not reaching 10 read count per million (CPM) in at least 4 RNA-Seq and 4 Ribo-Seq libraries were excluded from the analysis. Then, we performed the batch correction using ComBat-seq R package. ^30^ Principal component analysis (PCA) plots of the raw and batch corrected expression profiles are shown in Figure S3 in the *Extended data.* ^24^ A generalized linear model (glmQLFit, glmQLFTest of the edgeR package) was used to detect differentially expressed genes (for RNA-Seq, Ribo-Seq, and ribosome occupancy (RO) defined as the Ribo-Seq coverage of a CDS normalized to its RNA-Seq coverage) with the strain as a categorical variable. The false discovery rate (FDR < 0.05) was used for identification of differential expressed genes. We also performed Gene Ontology (GO) enrichment analysis for upregulated and downregulated genes with yeastmine. ^31^ The results are shown in Figure 1.

## Data availability

### Underlying data

NCBI Gene Expression Omnibus: RNA Sequencing and Ribosome profiling of *TMA46*, *STM1* and *YGR054W* knockout yeast strains. Accession number GSE185458; https://identifiers.org/geo:GSE185458.

NCBI Gene Expression Omnibus: RNA Sequencing and Ribosome profiling of GIR2 and PUB1 knockout yeast strains. Accession number GSE185286; https://identifiers.org/geo:GSE185286.

### Extended data

Figshare: Supplementary Table 1.csv https://doi.org/10.6084/m9.figshare.16818505. ^14^

This project contains the following extended data:

- Supplementary Table 1.csv (Table with information about sequencing experiments)

Figshare: Supplementary Figures. https://doi.org/10.6084/m9.figshare.16818610.v1. ^24^

Data are available under the terms of the Creative Commons Zero “No rights reserved” data waiver (CC0 1.0 Public domain dedication).
