# Supplementary material for: Ribo-Seq and RNA-Seq of TMA46 (DFRP1) and GIR2 (DFRP2) knockout yeast strains
Source: F1000Res. 2021 Nov 16;10:1162. [Version 1] doi: 10.12688/f1000research.74727.1 (PMC8637242; doi:10.12688/f1000research.74727.1)
Supplement: Peer review history

# Peer review: Reviewer response for version 1

**DOI:** 10.5256/f1000research.78509.r100404

**Published:** 2021-12-07

**Authors:** Nikolay E. Shirokikh, Katrina Woodward

**Affiliations:** 1 Division of Genome Sciences and Cancer, The John Curtin School of Medical Research, The Australian National University, Canberra, Australia; 2 Division of Genome Sciences and Cancer,The John Curtin School of Medical Research, The Australian National University, Canberra, Australia

The work by Artyom Egorov and others touches upon proteins implicated in the processivity of translation of mRNA into polypeptides by eukaryotic ribosomes, specifically in resolving the stalled (or ‘collided’) translational intermediates. Research in this area is accelerating with many new studies uncovering unforeseen depths of the cells’ mechanisms of translation fidelity control. Not too many datasets exist in this space to date and few are multi-target studies that provide a package of data obtained in a highly similar fashion for several translation-related genes, which is the rationale that justifies well the generated datasets.

 In a series of yeast knock-out strains, the authors accurately employ common approaches of RNA sequencing and RNA-sequencing-based ribosome (translation) footprint profiling, to provide high-throughput short-read sequencing data resulting from the individual gene knock-out effects. The data are provided for the wild-type yeast of BY4742 strain used as a control, the *GIR2 *and *TMA46 *knock-out mutant strains which are in the focus of the dataset, and additionally the *YGR054W* (eIF2A), *STM1,* and *PUB1* knock-out mutant strains, either commercially purchased or generated in the work.

 The methods are written succinctly but capture most of the relevant detail where it matters. Perhaps, expanding on the particulars of the method used to isolate mRNA with oligo(dT) beads could add to the better understanding of the RNA-seq data (including any information about the other treatments and purifications used, such as RNA fragmentation and size cut-offs). Regarding the ribosomal material isolation, although not strictly necessary, it would add to the value of the datasets by outlining their applicability if the authors could provide more detail on the intensity of the digestion with RNase I to generate the footprints, the homogeneity of the resultant sedimentation profiles, and the boundaries used to define the monosomal fraction. For the lysate clarification, the second spin uses very high acceleration; comments about whether the spin does or does not result in any loss of the heavy polysomes (e.g., if a suitably long particle sedimentation path was employed) could be beneficial. Although the authors are referring to the prior works, to bolster the applicability of the datasets and make their uptake convenient, any information about the additional size selection of the ribosome-protected fragments used (biochemical and/or bioinformatic) would be very helpful.

 The data appears as accurate. The authors provide some differential abundance and GO term enrichment information for the more and less abundant gene categories within the pairwise comparisons, to characterise the datasets. This is very useful information; more information regarding the general properties of the datasets (such as total read/mappable/unique read number tables or histograms, gene coverage saturation plots etc.) could be further attractive in the main figure. The replicated datasets are validated through principal component analysis plots in Supplementary Figure 3, which show generally good groupings between the replicated sample types. The validity of the ribo-seq data is confirmed by Supplementary Figure 1 through demonstrating triplet periodicity of the dominant-length footprint fraction usually attributed to the cycloheximide-stabilised ribosomal protection extent (28 nt; although this is shown only for the wild-type, *GIR2* and *TMA46* knock-out strains). The datasets are most definitely usable, presented accessibly including uploads to the NCBI Gene Expression Omnibus, and are of a high utility.

 Altogether, the authors provide an excellent resource to the field, which perfectly fits the scope of F1000Research Data Note publication, and will likely be cited.

Are sufficient details of methods and materials provided to allow replication by others?

Yes

Is the rationale for creating the dataset(s) clearly described?

Yes

Are the datasets clearly presented in a useable and accessible format?

Yes

Are the protocols appropriate and is the work technically sound?

Yes

Reviewer Expertise:

Molecular biology, biochemistry, cell biology, genetics and gene expression, RNA, protein biosynthesis, ribosome profiling

We confirm that we have read this submission and believe that we have an appropriate level of expertise to confirm that it is of an acceptable scientific standard.

# Peer review: Reviewer response for version 1

**DOI:** 10.5256/f1000research.78509.r100401

**Published:** 2021-12-01

**Authors:** Olivier Namy

**Affiliations:** 1 CNRS, I2BC, Université Paris-Saclay, Gif-sur-Yvette, France

In this manuscript Egorov A.A and colleagues aim to understand the translational consequences of the inactivation of two genes TMA46 and GIR2 in yeast *S. cerevisiae*. The experiments are clearly detailed and basic quality controls have been done for the RiboSeq analysis. It is not clear for me why the authors provided data for STM1, PUB1, and YGR054W without showing or analyzing them. I have several comments:

- The authors should justify why they have used a generalized linear model to calculate differential expression instead of a robust and well established negative binomial distribution (DESeq2). Why the authors used two different databases (silva-euk and rfam) to align reads to the rRNA? Did they use sequences specific to *S. cerevisiae*?
- Legend of Supplementary Fig1 needs to be improved. In the actual format, it is not obvious how the authors calculate the phasing of their RiboSeq data. Moreover, it is a pity that the analyses were not pursued a little further. For example, triplet periodicity could be shown, ribosome collision, and/or ribosome occupancy calculated. These additional qualitative analyses would strengthen the manuscript.

Are sufficient details of methods and materials provided to allow replication by others?

Partly

Is the rationale for creating the dataset(s) clearly described?

Yes

Are the datasets clearly presented in a useable and accessible format?

Yes

Are the protocols appropriate and is the work technically sound?

Yes

Reviewer Expertise:

NA

I confirm that I have read this submission and believe that I have an appropriate level of expertise to confirm that it is of an acceptable scientific standard.

# Peer review: Reviewer response for version 1

**DOI:** 10.5256/f1000research.78509.r100403

**Published:** 2021-11-30

**Authors:** Anton A. Komar

**Affiliations:** 1 Center for Gene Regulation in Health and Disease, Cleveland State University, Cleveland, OH, 44115, USA

In this manuscript, Egorov and co-authors presented transcriptome and ribosome profiling data for *Saccharomyces cerevisiae* strains with individual deletions of the *TMA46 *and *GIR2* genes. The interest in the protein products encoded by these genes has increased substantially in recent years, as these protein products have been shown to be involved in translation elongation and ribosome stalling. However, to date, there were limited “omics” data available from the respective knockout strains that could help shed additional light on the function of these genes. Particularly, ribosomal profiling of the knockout strains has not been performed. The present study fills in this gap and presents comparative ribosome profiling data obtained using the wild-type and knockout yeast strains. The ribosome profiling was followed by the standard quality checks and some basic downstream analysis, i.e. analysis of the differentially expressed genes and Gene Ontology (GO) enrichment analysis. Overall, these data will be useful for further research on TMA46 and GIR2 genes’ functions.

 The sample preparation was performed using the standard protocols. The raw and processed data were deposited in the relevant open access repositories such as the Gene Expression Omnibus (GEO). Data processing has been described with reasonable detail (including the description of the command-line tools options and adapter sequences). The reliability of Ribo-Seq data has been demonstrated by read length distribution and the corresponding fraction of reads in each frame (Supplementary figure 1). All in all, the data seem to be technically sound and accurately described, but I would suggest adding illustrative plots showing triplet periodicity of the ribosome footprints.

Are sufficient details of methods and materials provided to allow replication by others?

Yes

Is the rationale for creating the dataset(s) clearly described?

Yes

Are the datasets clearly presented in a useable and accessible format?

Yes

Are the protocols appropriate and is the work technically sound?

Yes

Reviewer Expertise:

Translational control of gene expression

I confirm that I have read this submission and believe that I have an appropriate level of expertise to confirm that it is of an acceptable scientific standard.
